# Network medicine: facilitating a new view on complex diseases

**DOI:** 10.3389/fbinf.2023.1163445

**Published:** 2023-05-24

**Authors:** Marija Cvijovic, Annikka Polster

**Affiliations:** ^1^ Department of Applied Mathematics and Statistics, University of Gothenburg, Gothenburg, Sweden; ^2^ Division of Systems and Synthetic Biology, Department of Life Sciences, Chalmers University of Technology, Gothenburg, Sweden

**Keywords:** complex diseases, network medicine, nosology, pathophysiology, multidimensional patient characterization

## Abstract

Complex diseases are prevalent medical conditions which are characterized by inter-patient heterogeneity with regards to symptom profiles, disease trajectory, comorbidities, and treatment response. Their pathophysiology involves a combination of genetic, environmental, and psychosocial factors. The intricacies of complex diseases, encompassing different levels of biological organization in the context of environmental and psychosocial factors, makes them difficult to study, understand, prevent, and treat. The field of network medicine has progressed our understanding of these complex mechanisms and highlighted mechanistic overlap between diagnoses as well as patterns of symptom co-occurrence. These observations call into question the traditional conception of complex diseases, where diagnoses are treated as distinct entities, and prompts us to reconceptualize our nosological models. Thus, this manuscript presents a novel model, in which the individual disease burden is determined as a function of molecular, physiological, and pathological factors simultaneously, and represented as a state vector. In this conceptualization the focus shifts from identifying the underlying pathophysiology of diagnosis cohorts towards identifying symptom-determining traits in individual patients. This conceptualization facilitates a multidimensional approach to understanding human physiology and pathophysiology in the context of complex diseases. This may provide a useful concept to address both the significant interindividual heterogeneity of diagnose cohorts as well as the lack of clear distinction between diagnoses, health, and disease, thus facilitating the progression towards personalized medicine.

## Introduction

Complex diseases are common, often chronic, non-communicable medical conditions characterized by a complex pathophysiology and etiology comprising of a combination of genetic and environmental factors. They are incompletely understood, but recent developments in the field of network medicine enable us to fundamentally reconceptualize them. Thus, network medicine facilitates a change in our approach towards disease conceptualization and study design by shifting focus from exploring diagnoses to understanding disease relevant traits and molecular features.

Complex diseases are found in all medical professions, and cover a broad spectrum of diagnoses, for example inflammatory disorders such as Crohn’s Disease and Rheumatoid Arthritis, neurodegenerative disorders such as Alzheimer’s and Parkinson’s disease, metabolic disorders such as Diabetes mellitus and Atherosclerosis, as well as various pain conditions such as Fibromyalgia.

Traditionally, such diagnoses have been defined based on clinical or histological presentation (e.g., sweet urine, loss of cognitive function) and not etiologically. In addition, disease classifications have been influenced by medical expertise being fragmented into medical specialties, in which complex diseases are identified and viewed through the lens of a predefined set of symptoms related to specific anatomical and histological structures.

Within complex disease diagnoses, the patient cohorts are notoriously heterogenous, and patients with the same diagnoses often show stark differences with regards to onset, symptoms profiles, trajectory, comorbidities, and treatment response. This heterogeneity, and the fact that disease etiologies are incompletely understood is a core reason why options for treatment and prevention remain suboptimal.

In this same tradition, comorbidities are usually conceptualized as distinct diseases, so essentially several simultaneously active distinct pathomechanisms, which thus need to be addressed separately. With our increased understanding of the intricate mechanisms underlying complex diseases, it is becoming clear that not only are comorbidities relevant for treatment response and -satisfaction, but also that seemingly distinct conditions may be more connected than previously thought, and that we need new concepts that enable us to better research, understand and treat complex diseases.

Network medicine fills two roles in ameliorating the challenges of complex diseases. For one, the insights gained in recent studies allow us to fundamentally change our mental model of complex diseases. In addition, the field provides an ever-growing body of tools to understand human pathophysiology in an unmatched comprehensiveness. To make the most of these opportunities, we need to reciprocally update the nosology based on new knowledge and redesign our research questions based on these new models.

## What is network medicine teaching us?

The field of network medicine explores disease pathophysiology by generating complex networks. These networks can represent associations between biological components on a molecular or genetic level, or associations between disease characteristics (Barabási et al., 2011; Sonawane et al., 2019). This approach facilitates a more comprehensive understanding of the various factors relevant in human diseases and their underlying pathomechanisms.

Among the many novel insights network medicine has provided in the recent years, three lessons may have the most impact on reconceptualizing complex diseases. The first lesson was that many current diagnoses are not clearly distinct entities but frequently share mechanisms (Goh et al., 2007; Halu et al., 2019), overlap in their clinical presentations (Hoehndorf et al., 2015) and show patterns of co-occurrence (Amell et al., 2018). The second lesson highlighted the complexity of individual pathophysiological mechanisms by showing the poly-to omnigenic factors involved in cell and tissue differentiation (Boyle et al., 2017; Sonawane et al., 2017) and the complexity across multiple levels of biological organization (Halu et al., 2019). The third lesson showed that diagnoses which share genetic components also tend to share symptoms (Halu et al., 2019; Sánchez-Valle et al., 2020).

With regards to complex diseases, these results illustrate substantial pleiotropy embedded in complex pathomechanistic systems and thus highlight the heterogeneity throughout the disease space. In addition, they suggest individual pathophysiology that is so multifactorial that it is near-impossible that two individuals share the exact same mechanisms, but also that features relevant to some individuals pathomechanisms are shared by others with a completely different clinical presentation, or even with healthy individuals.

## Rethinking complex diseases

In light of these findings, the current nosological classifications present as incommensurate to the multidimensional disease pathogenesis that is inherent to complex diseases. The use of these diagnostic classifications may even inhibit an efficient progression towards personalized and precision medicine as using them as inclusion/exclusion criteria or outcome measures in studies may be the origin of a substantial amount of noise in the respective datasets. Genome-wide association studies for example typically utilize disease diagnoses as phenotype measure. If the respective diagnosis is heterogenous, i.e., an umbrella for a group of patients with various endotypes, the resulting associations may suffer from substantially inaccurate conclusions. It is therefore time to rethink our view of complex diseases and identify more useful concepts than that of multifactorial but distinct entities.

One option for reconceptualizing the bulk of complex diseases would be a model of overlapping clusters of clinical phenotypes, where the different phenotypes within these clusters are non-identical and determined by multifactorial mechanisms. While being a big step forward, this approach fails to adequately address comorbidity patterns.

When aiming to better understand clinical phenotypes, it is important to reflect on symptom expression and symptom interpretation. In line with thinking of complex traits being determined by omnigenic interactions, one may argue that symptoms are themselves a function of a large number of traits. Pain for example can be determined by nociceptor characteristics, local and global immune status, afferent and efferent neural signaling and central processing, as well as sociocultural and psychological factors, and likely by many more (latent) traits. Additionally, while diagnoses are based on a set combination of symptoms (including biomarkers, imaging etc.), the disease burden of an individual patient goes beyond that and may be defined as the function of the comprehensive set of symptoms of the individual. This individual disease burden may be more tightly tied to the respective patients endotype than a diagnose attributed to them, and thus it is likely beneficial to be represent the individual disease burden in more detail in many study designs.

**FIGURE 1:**
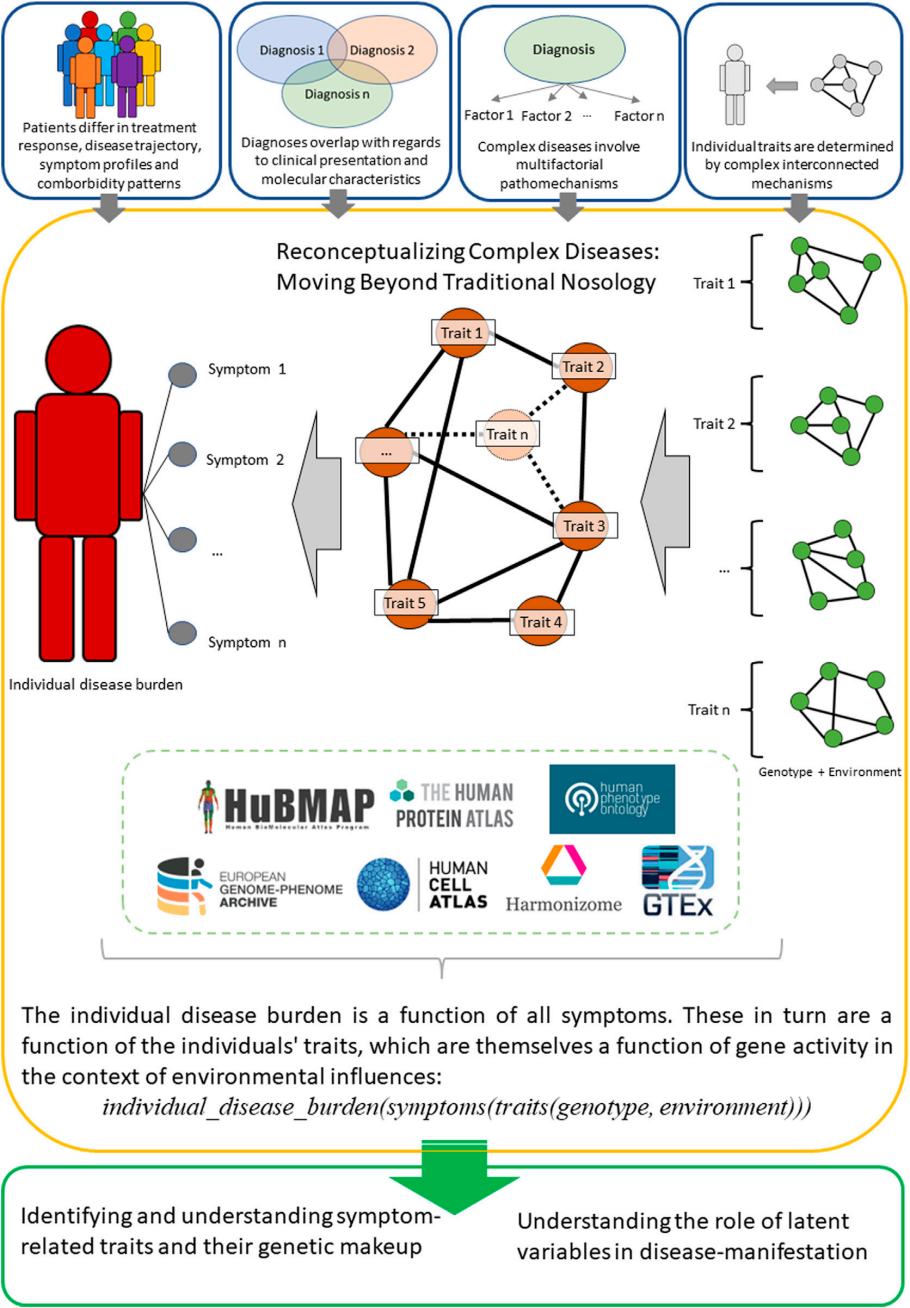
An overview of the proposed reconceptualization of complex diseases, which instead of traditional diagnoses focuses on individual disease burdens as a function of multidimensional features. Set of selected databases (not a comprehensive list) is an optional entry-point that could serve for a preliminary analysis and to facilitate novel experiments.

Taken together, a conceptualization that aims to generate a comprehensive representation of a patient’s phenotype and endotype may be necessary to progress our understanding of complex diseases. A potential way of doing this would be to focus on individual disease burdens, defined as a function of the symptoms the individual presents (Figure 1). As described above, these symptoms would in turn be a function of (latent) traits, which in themselves are a function of the individuals gene expression in the context of transcription-modifying environmental factors.

One possibility of representing such a concept would be a via a state vector, in which the person-specific individual disease burden is defined as a function of the symptoms presented by the respective person: *individual_disease_burden(symptoms*).

The symptoms themselves are in turn considered not as single traits but as the result of a combination of the traits of an individual: *symptoms(traits*).

These traits can subsequently be understood as a function of genetic features in the context of environmental factors which alter transcription: *traits(genotype, environment*).

Taken together, this results in the following formalization, where state vectors are utilized as descriptors of molecular and physiological characteristics, thus allowing for a high-dimensional representation of individual disease burden: *individual_disease_burden*(*symptoms*(*traits(genotype, environment)*)).

This framework offers multiple relevant opportunities, including 1) the ability to approximate the burden of disease for an individual patient in comparison to similar patients 2), relate different levels of biological organization to these disease burdens, and 3) incorporating comorbidities into the patient characterization process in an automated manner. Thus, this conceptual model would facilitate understanding of human pathology in the context of personalized disease representations, and aid in overcoming reductionist diagnostic confinements. The most challenging part of this representation is to identify a comprehensive set of symptom-defining traits. In this context, the nested representation can reflect the current body of knowledge, where individual genetic and transcriptomic characteristics as well as symptom presentation can be measured. The level of traits, and subsequently endotypic characteristics, are then represented as latent variables. Using this approach, the focus of future study designs can then be shifted from diagnoses to identifying and understanding symptom-related traits and their genetic makeup.

Community efforts to collect and systematically organize available data resulted in a large number of databases (Table 1). The data available in such databases includes disease-related phenotype data, drug response as well as genomic, transcriptomic and spatial features of tissues and cells, with single-cell data becoming more and more available. These databases can be used both as guidelines for further experiments and as a basis to identify key players in networks of interest and identify connections between various levels of biological organization.

**TABLE 1:** A selection of projects that collect data relevant for research on human phenotypes and molecular features.

Database	Brief description
Human Protein Atlas (1)	The Human Protein Atlas aims to comprehensively map all human proteins within cells, tissues, and organs. Multiple omics technologies are utilized and integrated. The datasets include antibody-based imaging, proteomics, transcriptomics, and systems biology. https://www.proteinatlas.org/
Genotype-Tissue Expression project (2)	The Genotype-Tissue Expression (GTEx) project aims to create a comprehensive open-access database for investigating tissue-specific gene expression and regulation. Samples were collected from nearly 1,000 individuals across 54 non-diseased tissue sites, and mainly analysed through whole-genome sequencing, whole-exome sequencing and RNA sequencing. https://www.gtexportal.org/home/
Human Phenotype Ontology (3)	The Human Phenotype Ontology (HPO) gives a standardized vocabulary of phenotypic abnormalities encountered in human disease. https://hpo.jax.org/app/
	To facilitate the utilization of the HPO a bioinformatics tool called the Phenomizer (4) is available to investigate phenotype data. It uses a semantic similarity algorithm to calculate the similarity between the patient’s phenotype and the phenotypes associated with different diseases. https://hpo.jax.org/app/tools/phenomizer
Harmonizome (5)	The Harmonizome is a database and search engine created to explore relationships among genes, proteins, and other biological entities. It integrates over 100 datasets and includes gene expression, protein-protein interactions, epigenetics, and more. https://maayanlab.cloud/Harmonizome/
Human BioMolecular Atlas Program (6)	The Human BioMolecular Atlas Program (HuBMAP) is an ongoing initiative to create an open and global atlas of the human body at the single-cell level by mapping various tissues and organs using different technologies such as spatial transcriptomics and proteomics. https://portal.hubmapconsortium.org/
The European Genome-Phenome Archive (7)	The European Genome-phenome Archive (EGA) is a resource for sharing and accessing human genetic and phenotypic data generated by various research studies. https://ega-archive.org/
The Human Cell Atlas (8)	The Human Cell Atlas (HCA) is an ongoing initiative that utilizes genomics, transcriptomics, proteomics, and spatial imaging to create a high-resolution map of all the cells in the human body. http://humancellatlas.org/

This table is intended to provide an entry-point but cannot be a comprehensive list of all data repositories.

^1^ Uhlén M, Fagerberg L, Hallström BM, et al. Proteomics. Tissue-based map of the human proteome. *Science.* 2015; 347 (6220):1260419. doi:10.1126/science.1260419.

^2^ GTEx Consortium. The Genotype-Tissue Expression (GTEx) project. *Nat Genet.* 2013; 45 (6):580–585. doi:10.1038/ng.2653.

^3^ Köhler S, Gargano M, Matentzoglu N, et al. The Human Phenotype Ontology in 2021. *Nucleic Acids Res.* 2021; 49 (D1):D1207-D1217. doi:10.1093/nar/gkaa1043.

^4^ Köhler S, Schulz MH, Krawitz P, et al. Clinical diagnostics in human genetics with semantic similarity searches in ontologies. *Am J Hum Genet.* 2009; 85 (4):457–464. doi:10.1016/j.ajhg.2009.09.003.

^5^ Rouillard AD, Gundersen GW, Fernandez NF, et al. The harmonizome: a collection of processed datasets gathered to serve and mine knowledge about genes and proteins. *Database (Oxford).* 2016; 2016:baw100. Published 2016 July 3. doi:10.1093/database/baw100.

^6^ HuBMAP Consortium. The human body at cellular resolution: the NIH Human Biomolecular Atlas Program. Nature. 2019; 574 (7777):187–192. doi:10.1038/s41586-019-1629-x.

^7^ Freeberg MA, Fromont LA, D’Altri T, et al. The European Genome-phenome Archive in 2021 [published correction appears in Nucleic Acids Res. 2023 April 11; 51 (6):2994]. Nucleic Acids Res. 2022; 50 (D1):D980-D987. doi:10.1093/nar/gkab1059.

^8^ Regev A, Teichmann SA, Lander ES, et al. The Human Cell Atlas. Elife. 2017; 6:e27041. Published 2017 December 5. doi:10.7554/eLife.27041.

An additional consideration here is the treatment response of an individual patient. As outlined above, many complex diseases are difficult to treat as treatment response differs between patients with the same diagnoses. In the nested model presented here, individual treatment response can also be viewed as a function of disease-relevant traits. By using treatment response in such a way, especially when analyzing data from patients that have tried different treatment options, this characteristic can then be utilized as a steppingstone to narrow down relevant latent traits and their role in disease manifestation.

## Discussion

The mind-boggling intricacies that are inherent to the nature of complex diseases, including various levels of biological organization as well as environmental and psychosocial factors, make these disorders difficult to study, understand, prevent, and treat. Given our increased understanding of mechanisms that are shared between diagnoses and patterns of co-occurrence, our traditional conceptualization of complex diseases, where diagnoses and comorbidities are seen as distinct entities, seems outdated. To reduce statistical noise in research projects and facilitate knowledge gain and the progression towards personalized and precision medicine, we thus need to find better ways of conceptualizing complex diseases and the complex phenotypes of individual patients.

By reframing our concepts and shifting our view away from diagnoses towards individual disease burdens as a function of the individuals’ symptoms and then aim to understand symptom-relevant traits, we may be able to better address the aforementioned challenges and improve the quality of our research data.

Undoubtedly, this approach also has limitations. It is difficult to measure symptoms, even more difficult to attempt to measure a comprehensive set of symptoms, and likely impossible to measure a comprehensive set of traits. Additionally, methods for adequately dealing with high-dimensional phenotype data are scarce. There are currently no good solutions to these limitations, but they nevertheless need to be regarded and addressed to identify workarounds and develop new tools. With this contribution we hope to suggest a novel models and spark discussions to establish better concepts to address the limitations of our current nosological and epidemiological definitions. By refining the presented ideas and exploring them using real-life data novel insights into human pathophysiology may be established and a progression towards personalized medicine facilitated.

## Data Availability

The original contributions presented in the study are included in the article/supplementary material, further inquiries can be directed to the corresponding author.
